# How does venture capital cross-border syndication spur corporate innovation? Evidence from China

**DOI:** 10.3389/fpsyg.2022.921168

**Published:** 2022-07-22

**Authors:** Haixia Hao, Lihong Guo, Jianwei Dong

**Affiliations:** School of Economics and Management, Northwest University, Xi’an, China

**Keywords:** venture capital, cross-border syndication, corporate innovation, cross-border quadratic relationship closure, strategic cooperation relationship

## Abstract

In recent years, venture capital (VC) cross-border syndication has shown an obvious growth trend. Based on the existing studies, this paper explores the impact of VC cross-border syndication on corporate innovation. We also examine the mediating roles of cross-border quadratic relationship closure (CBQRC) formed by the strategic cooperation relationship between the respective portfolio companies of domestic and foreign VCs. This paper conducted an empirical analysis to test our hypotheses using a sample of first-round investments in domestic firms by domestic VC firms from 2014 to 2016. Results show that the more investment events of VC cross-border syndication or the more partners of VC cross-border syndication, the more likely it is to have a significant positive impact on the innovation of domestic portfolio companies. CBQRC plays a mediating role between VC cross-border syndication on corporate innovation. Results remain robust after removing endogeneity using the instrumental variables approach and removing sample selection bias using Heckman two-stage regression. Results deepen the understanding of the relationship between VC cross-border syndication and corporate innovation and provide essential guidance to domestic VC firms promoting corporate innovation in open partnerships.

## Introduction

Technological innovation is an activity of high cost, uncertainty, and risk, because of the information asymmetry between the owners of startups and external investors (Binks and Ennew, 1996), which makes it difficult for startups to obtain the capital of banks and other financial intermediaries for corporate innovation. In addition, startups can only generate limited cash flow (Arvanitis and Stucki, 2012) and can hardly afford the high R&D costs. As a result, capital constraints become the biggest problem that prevents startups from crossing the “valley of death” period when they intend to carry out innovative activities. At this stage, startups have minimal access to capital, and venture capital (VC) is a critical force in helping startups across the “valley of death” and then grow quickly. VC invests in unlisted and high-growth startups through equity investments (Han, 2021), and successfully exits startups by means of mergers and acquisitions (M&A) or initial public offerings (IPO) to obtain considerable returns (Zheng, 2022). VC is widely credited with supporting the development of global high-tech industries because of its ability to provide critical support for startups’ early survival and growth (Hirukawa and Ueda, 2011). Many world-class innovative companies, such as Apple, Facebook, and Alibaba, have received VC support in the early stage of development. Therefore, VC plays a considerable role in promoting innovation and entrepreneurial activities.

Take China’s VC market as an example. Since the Chinese government proposed the development policy of mass entrepreneurship and innovation, the enthusiasm for innovation and entrepreneurship in China has never been higher, and with the emergence of a large number of high-quality entrepreneurs, more and more investment institutions and investors have started to pay more attention to and support the development of innovation. Lin (2017) shows that China’s VC market provides a case worth studying due to the fact that China has become the second-largest VC market in the world, showing a rapid growth in terms of fundraising, financing amount, and exit channels for capital. In addition, according to a recent report by PitchBook, China’s total VC investment reached $113.8 billion in 2021, ranking second in PitchBook’s data records and near an all-time high.^1^ Therefore, China’s VC market becomes an ideal context to study the impact of VC on entrepreneurial and innovative activities.

The VC industry has long been a local industry (Cumming and Dai, 2010) because geographical proximity to portfolio companies allows for effective monitoring and value-added services (Mäkelä and Maula, 2006). However, with the increased competition in the domestic VC market, more and more VC firms are moving out of the country to look for investment opportunities abroad (Chemmanur et al., 2016). In the operational practice of domestic VC, cross-border syndication with foreign VC is a frequent investment approach adopted by domestic VC. VC firms in syndication have different skills, and they can comprehensively supervise, constrain, and evaluate the development of startups by complementing each other’s advantages (Bayar et al., 2020). Therefore, cross-border syndication with foreign VC has become the primary form for domestic VC to actively integrate into the global innovation network. According to the statistics provided by Crunchbase database, domestic VC cross-border syndication has developed rapidly in recent years. The number of investment events of domestic VC cross-border syndication was only 317 before 2013, while it reached 1,985 from 2014 to 2019. Although the rise of domestic VC cross-border syndication brings the advantages of risk diversification and opinion assistance, the impact of domestic VC cross-border syndication on corporate innovation is still under-revealed due to the agency problem caused by information asymmetry and development uncertainty of startups. Therefore, it is necessary to study the impact of VC cross-border syndication on corporate innovation.

Based on the related research of VC post-investment management (Wang et al., 2012; Ozmel et al., 2013a,b), VC syndication (Sorenson and Stuart, 2001, 2008; Meuleman et al., 2017; Zhelyazkov, 2018) and corporate cooperative innovation (Luo, 2002; Belderbos et al., 2004; Fitjar and Rodríguez-Pose, 2013; Hsieh et al., 2018; Yang et al., 2018), this study proposes a new mechanism, namely, cross-border quadratic relationship closure (CBQRC), to reveal the role of domestic VC in promoting domestic corporate innovation through cross-border syndication. The core idea of CBQRC is summarized: Domestic VC cross-border syndication can help their portfolio companies establish strategic cooperation relationships with cross-border partners’ foreign portfolio companies, thus promoting innovation of domestic portfolio companies. By empirically analyzing a sample of first-round investments in domestic companies by domestic VC firms from 2014 to 2016, we obtained the following conclusions: (1) The proactive integration of domestic VC into global innovation networks through cross-border syndication can significantly enhance corporate innovation. (2) CBQRC plays a mediating effect in the impact of domestic VC cross-border syndication on corporate innovation.

## Literature review

### Venture capital cross-border syndication

Venture capital cross-border syndication refers to multiple VC firms collaborating across geographical boundaries to participate in the same investment activity, providing the required resources and sharing the investment results (Lerner, 2000; Mäkelä and Maula, 2006). More and more VC firms have started to go abroad for cross-border syndication in recent years, which has aroused great interest among scholars. Many scholars have examined VC cross-border syndication from multiple perspectives, and basically, these existing studies can be summarized in two aspects: risk sharing and value-added.

On the one hand, VC is a high-risk investment activity, especially in cross-border investment. The geographical distance, institutional distance, and cultural distance between VC firms and their portfolio companies make the information asymmetry dilemma more obvious (Chemmanur et al., 2016). Although foreign VCs have advantages in resources and expertise, they also have disadvantages in terms of local knowledge and networks that affect investment performance (Mäkelä and Maula, 2008). Therefore, when domestic VC firms go abroad to invest in overseas markets, cooperation with local VC firms in foreign countries can help domestic VC firms gain access to local knowledge and resources and help reduce information asymmetry. In addition, when multiple investors participate in an investment activity together, each VC firm can use less capital to invest in more areas of interest and achieve the purpose of risk diversification (Khavul and Deeds, 2016).

On the other hand, in their role as value-added service providers, VC firms have access to detailed information about the strategies and development dynamics of the company. They can use this information to identify profitable collaborations between companies (Lindsey, 2008). Different VC firms may have different strengths in terms of connections, capital, and social networks (Brander et al., 2002), so that they can guide companies in their innovation activities more comprehensively and provide resources in various areas of expertise to their portfolio companies, thus avoiding abortive innovation activities due to lack of industry experience and expertise. In addition, VC firms can also learn from other partners through cross-border syndication to make up for their internal knowledge deficiencies and improve investment performance (Khurshed et al., 2020). Therefore, from the perspectives of risk sharing and value addition, it can be found that VC cross-border syndication is beneficial to reducing risk and integrating entrepreneurial resources to help startups grow.

### Cross-border quadratic relationship closure

Factors influencing partner selection have long focused on organizational theorists studying partnerships, including strategic alliances (Mitsuhashi and Greve, 2009) and VC syndication (Sorenson and Stuart, 2008; Plagmann and Lutz, 2019). Granovetter (1973) described the phenomenon of two strangers creating strong and weak ties through some common medium as a closed triad. Based on this idea, Kossinets and Watts (2006) described the process of two strangers getting to know each other through a third person as triadic closure, and their study showed that the role and status of the third person had a strong influence on the strength of the relationship between the other two. Lindsey (2008) explores the phenomenon of triadic closure in VC syndication, where they find that the likelihood of two startups forming an alliance increases if the two startups have a common VC firm. Thus, triadic closure has been documented in many empirical settings, particularly in clusters of relationships that tend to develop intensive, interconnected relationships (Gulati et al., 2012; Zhelyazkov, 2018).

**FIGURE 1:**
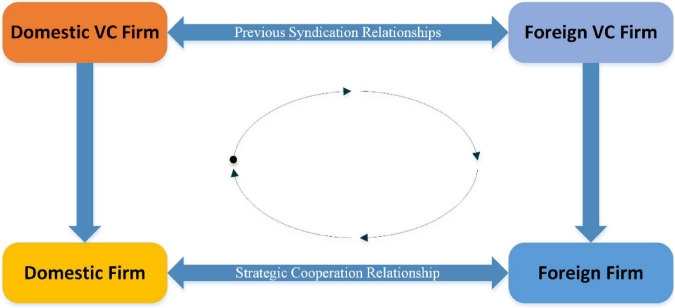
Structure of the cross-border quadratic relationship closure.

To date, however, limited attention has been paid to the downstream relationship between VC firms and their portfolio companies. Although existing literature has focused on how ties to a shared third party can affect the outcome of the relationship between two organizations, scholars have overlooked the importance of closure in a partnership in facilitating or inhibiting direct collaboration between indirectly linked actors of two organizations. For example, On 23 May 2017, a China company—Realtime Technology announced a strategic partnership with a US company-Immersion, planning to apply Immersion’s technology in Realtime Technology’s products. Before these two companies formed a strategic partnership, Realtime Technology had received a Series A investment from Tencent Capital on 11 April 2016, and Immersion had received a Series A investment from Intel Capital on 24 March 2011. In addition, Tencent Capital and Intel Capital had jointly invested in Ark, a US-based search engine company, on 25 April 2012. Combining this VC investment event case, we can find interconnection among Realtime Technology (Domestic firm)—Tencent Capital (Domestic VC firm)—Intel Capital (US VC firm)—Immersion (US firm) form a CBQRC, as shown in Figure 1. Before Tencent Capital and Intel Capital co-invested, perhaps Realtime Technology and Immersion did not know each other, but after the two VC firms co-invested across the border, it is possible to increase the possibility of their acquaintance and cooperation.

The formation of CBQRC creates opportunities for domestic and foreign VC firms’ respective portfolio companies to be more likely to establish direct collaboration with each other (Rogan and Sorenson, 2014). In addition, the involvement of domestic and foreign VCs in startups also releases relevant signals about the quality of the startups to the market to some extent, helping to alleviate any concerns of the partners about the capabilities and motivations of the startups themselves (Zhelyazkov, 2018), making the stability, trust, and benefits of this pluralistic relationship more likely to arise (Li and Piezunka, 2020). However, as with most partnerships, the stability of collaborative relationships between portfolio firms can be challenged by some uncertainty. For example, Pahnke et al. (2015b), by investigating the impact of early relationships on innovation in entrepreneurial firms, find that competitive information leakage occurs when firms are indirectly linked to competitors through shared intermediary organizations, which can hinder young firms’ innovation efforts and reduce the effectiveness of collaboration. Related studies have also shown that achieving successful collaboration between different organizations means facing the challenge of coordination and communication among multiple parties (Gulati et al., 2012), such challenges change even more in the context of cross-national collaboration. These challenges include poor language communication, institutional and cultural differences, disagreements, and conflicts, all of which can undermine interorganizational collaborative efforts and make collective success difficult to achieve (Gulati et al., 2008; Wang et al., 2022). Therefore, the successes and challenges in strategic cooperation among portfolio companies in the context of VC internationalization prompt further academic research on the evolution and outcomes of cooperation among relationship subjects.

### The impact of venture capital cross-border syndication on corporate innovation

Previous research has shown that VC firms’ involvement in startups helps portfolio companies find strategic partners (Lindsey, 2008). Similarly, the more VC firms invest in a given firm or the more rounds of financing, the more strategic partners the firm is likely to acquire (Wang et al., 2012; Ozmel et al., 2013a). In particular, when startups have multiple investors, the more prominent the VC firm is in that network of relationships, the more likely the portfolio company is to form R&D partnerships with established firms (Ozmel et al., 2013b). Therefore, VC involvement can have a profound impact on the subsequent development of a startup.

Innovation, as an important part of a company’s core competence, is an essential indicator for investors to assess the value of a company and reflects its market value. According to previous research, Lemley (2001) observed that venture capitalists use client patents (or more likely patent applications) as evidence that the firm is well-managed, at a particular stage of development, and has identified and developed a market niche. Similarly, Hsu and Ziedonis (2013), using a sample of 370 VC-involved startups, find that firms with more patents can receive more dramatic valuation adjustments when they go public. These quotes imply that VCs focus on the patenting activities of startups to monitor the firms’ innovation process and promote the firms’ use of innovation advantages to improve their market position. Based on the above analysis, we believe that VCs will pay attention to firms’ patent activities when participating in startups.

Based on the existing literature on the relationship between VC and corporate innovation and the inconsistency of existing research findings on the relationship between the two, we argue that the relationship between VC cross-border syndication and corporate innovation deserves further study, especially in the absence of existing research on the Chinese VC ecosystem. In fact, startups’ high degree of uncertainty makes it very difficult to obtain funds from external investors. However, VC investment in startups can help promote innovation by providing more funds for R&D activities and solving the dilemma of difficult and expensive financing for startups. Moreover, VCs’ participation in startups can signal to the market that the startups are of high quality, reduce the information asymmetry between startups and external investors, and help moderate the subsequent financing costs. In addition, the ultimate goal of VC is to successfully exit the portfolio companies through IPO and M&A after the value of the startups has increased, in order to earn excess investment income. Therefore, as a professional investment institution, VCs are motivated to pay attention to and participate in the R&D decisions of their portfolio companies to improve their innovation capabilities for their own investment returns.

In the context of cross-border syndication, it has been shown that domestic and foreign VC firms that have experienced cross-border syndication are more likely to repeat the syndication in the future (Zhelyazkov, 2018). Furthermore, classical literature also shows that cooperation with other firms is beneficial for firm innovation (Luo, 2002), especially with non-local firms (Belderbos et al., 2004). Similarly, some scholars further found that cooperation with foreign firms helps domestic firms to innovate (Fitjar and Rodríguez-Pose, 2013; Hsieh et al., 2018), especially when there is mutual trust between the two partners (Yang et al., 2018). Taken together, we argue that the VC cross-border syndication will improve the innovation of domestic portfolio companies. Based on the above analysis, the following hypotheses are proposed in this paper.

**Hypothesis 1:** VC cross-border syndication will have a positive impact on the innovation of domestic portfolio companies.

### Mediating role of cross-border quadratic relationship closure

Related studies have shown that interorganizational cooperation tends to produce achievements beyond what any single organization can achieve (Wang et al., 2022). Factors such as complementary capabilities, similarity in domain specialization, etc. can predispose two organizations to cooperate (Sorenson and Stuart, 2008; Shipilov and Li, 2012). Applied to the research context of this paper, we argue that the strategic partnerships formed in the process of cross-border cooperation between domestic and foreign firms will create long-term and sustainable value for both parties by leveraging their expertise and industry resources. In addition, cooperation between domestic and foreign firms linked through domestic and foreign VC firms also has the potential to reduce costs, secure supply chains, reduce competition, increase resources (Chang, 2004), and create other synergistic effects.

Therefore, the CBQRC formed by the strategic cooperation relationship between the respective portfolio companies of domestic and foreign VCs creates a bridge between VC cross-border syndication and the innovation of their portfolio companies. The formation or not of strategic partnership determines the relationship closure between the four innovation agents (domestic firm—domestic VC firm—foreign VC firm—foreign firm). When domestic and foreign firms are linked by having cooperated with domestic and foreign VCs, the formation of CBQRC helps provide legitimacy to their portfolio companies, reduces search costs for resource-poor new ventures, and reduces expropriation problems by monitoring and penalizing non-cooperation (Wang et al., 2012). In addition, from the perspective of alliance formation, VC firms use their expertise to manage information flows and identify profitable alliance opportunities (Lindsey, 2008). After forming an alliance, VC firms in a syndicate provide a broader range of value-added services to their respective portfolios through complementary management skills and shared social capital (Brander et al., 2002). Thus, domestic and foreign portfolio companies are more likely to benefit from the different information, expertise, and network relationships that different VC firms have through prior collaboration, which in turn affects the portfolio companies’ innovation. Based on the above analysis, we argue that VC cross-border syndication helps domestic portfolio companies establish strategic cooperation partnerships with foreign portfolio companies invested by foreign VC firms by forming a CBQRC, thus influencing the innovation of domestic portfolio companies. The following hypotheses are proposed in this paper.

**Hypothesis 2:** Cross-border quadratic relationship (CBQRC) plays a mediating role in the impact of VC cross-border syndication on the innovation of domestic portfolio companies.

## Research design

### Data sources and sample selection

The data sources used in this paper are shown as follows. First, the data on domestic VC investment events and the characteristics of domestic VC comes from the Private Equity Database. Second, the number of cross-border syndication events and cross-border syndication partners of domestic VC comes from Crunchbase Database. Third, the data on corporate innovation are collected through the China Intellectual Property Right Net. Fourth, the data on strategic cooperation between domestic firms and foreign firms is collected through their official websites and the Baidu search engine. Finally, the data on the director assignment and CEO replacement of firms are collected through the Tianyancha Database. It is remarkably, however, that although the Private Equity Database is widely used in China’s VC research, it is a domestic commercial database with poor coverage of domestic VCs cross-border syndication events and their cross-border syndication partners’ investment events. In contrast, Crunchbase database, as a global international database, covers foreign VCs investment data worldwide, which can solve the data shortage problem of Private Equity database. Combining those two databases can interoperate and improve data coverage and data quality.

This paper obtained a sample of domestic VC firms’ first-round investments in domestic companies from 2014 to 2016 through the Private Equity Database. The starting point of the sample is 2014 because there were few domestic VC cross-border investment events before that, and the data become quite comprehensive after that year in time. We use 2016 as the sample termination point is required to retain the first post-investment year as the observation period for VC post-investment management, including the director assignment, CEO replacement, and the establishment of strategic cooperation partnerships between domestic firms and foreign firms, and the second and third post-investment years as the observation period for the innovation of domestic portfolio companies. For example, Legend Capital invested in Lanchai, a Beijing-based Fintech company, on 1 January 2015. Then the period for observing Legend Capital’s post-investment management is from 2 January 2015 to 1 January 2016 (the first year after VC investment), and the period for observing Lanchai’s innovation activities is from 2 January 2016 to 1 January 2018 (the second and third years after VC investment). After excluding companies with undisclosed key information, we finally obtained a final sample of 1,311 VC first-round investments with complete information.

### Variables

#### Explained variable

The types of patents are regulated differently in different countries. China’s patent law classifies patents as invention, utility model, and design patents. Zhou et al. (2017) argue that invention patents are a better indicator of corporate innovation than utility model patents and design patents. Therefore, following the common practice in the international literature, this paper uses the number of granted invention patents to measure corporate innovation.

#### Explanatory variable

This paper observes the active integration of domestic VC into global innovation networks through cross-border syndication from two perspectives: (1) Number of investment events of domestic VC cross-border syndication; (2) Number of partners of domestic VC cross-border syndication. When counting these two indicators, there are two points to note. First, the domestic VC cross-border syndication here refers specifically to domestic VC firms co-investing in foreign companies with foreign VC firms in the same round, and the sample of purely domestic VC firms co-investing in foreign companies with other domestic VC firms in the same round is not included. Second, the cross-border syndication partners of domestic VC firms include both current and previous cross-border syndication partners.

#### Mediation variable

Most previous studies have relied on sizeable strategic alliance databases to observe strategic partnerships among firms. For example, Wang et al. (2012) used the SDC Alliances database, Ozmel et al. (2013a) used Recombinant Capital’s Strategic Alliance database, and Ozmel et al. (2013b) used the Deloitte Recombinant LLC database. It is reliable to use large commercial databases to observe strategic cooperation partnerships between enterprises, but unfortunately, there is no similar database in China, and these foreign databases mentioned have a poor coverage of strategic cooperation partnerships between Chinese companies. Therefore, we developed a multistage procedure to observe whether the domestic portfolio companies invested by domestic VCs and the foreign portfolio companies invested by foreign VCs established a strategic cooperation partnership. First, we searched the names of all the foreign strategic cooperation partners of the domestic portfolio companies invested by domestic VCs through their official websites. Second, we identified the timing of the formation of the strategic partnership through the Baidu search engine. Finally, we use the Crunchbase database to determine the investors of the foreign strategic cooperation partners. If the investors of those foreign strategic cooperation partners include domestic VC firms’ cross-border syndication partners, it is defined as a CBQRC and takes the value of 1. If the investors do not include domestic VC firms’ cross-border syndication partners or domestic portfolio companies do not establish strategic partnerships with foreign portfolio companies, it is defined as non-CBQRC and takes the value of 0.

#### Control variables

Drawing on existing research and data availability, this paper selects a series of control variables regarding VC level and firm level, respectively. The specific definitions of the control variables are shown in Table 1.

**TABLE 1:** Variable descriptions.

	Variables	Index	Definition
Explained variable	*CI*	Corporate innovation	The number of invention patents granted by domestic portfolio companies in the second and third years after receiving VC investment.
Explanatory variable	*Ne*	Number of investment events of domestic VC cross-border syndication	Cumulative number of investment events in which domestic VC cross-border syndication prior to investing in domestic companies.
	*Np*	Number of partners of domestic VC cross-border syndication	Cumulative number of partners of domestic VC cross-border syndication prior to investing in domestic companies.
Mediation variable	*CBQRC*	Cross-border quadratic relationship closure	The value is 1 if the domestic VC’s domestic portfolio company establishes a strategic cooperation partnership with a foreign company invested by foreign VC, who are cross-border syndication partners of domestic VC within the first year after receiving the investment, 0 otherwise.
Control variable	*Se*	Successful exits	The cumulative number of domestic VC successful exits through M&A and IPO prior to investing in domestic companies.
	*IPO*	IPO on foreign stock exchanges	The cumulative number of domestic VC exits through IPOs on foreign stock exchanges prior to investing in domestic companies.
	*Rep*	VC reputation	In the year of VC investment in domestic companies, 1 if the VC is listed in the annual ranking list of China equity investment published by Zero2IPO Group, 0 otherwise.
	*Sob*	State-owned background	1 if the VC has a state-owned background, 0 otherwise.
	*Syn*	VC syndication size	When VC invests in domestic companies, 1 if the number of investors is greater than or equal to 2, 0 otherwise.
	*Da*	Director assignment	Within the first year after the domestic portfolio company receives investment from domestic VC, 1 if domestic VC assigns directors to the domestic portfolio company, 0 otherwise.
	*CEO*	CEO replacement	Within the first year after the domestic portfolio company receives investment from domestic VC, 1 if domestic VC replaces the CEO of the domestic portfolio company with an experienced external CEO, 0 otherwise.
	*Pci*	Previous corporate Innovation	The total number of invention patents, utility model patents, and design patents applied for by domestic portfolio companies in the five years prior to receiving domestic VC investment.
	*Tech*	Hi-tech dummy	1 if domestic portfolio company belongs to the high-tech industry, 0 otherwise.
	*Early*	Early dummy	1 if development stage of the domestic portfolio company is in the seed stage or start-up stage, 0 otherwise.
	*Age*	Company age	The difference in years between the year the domestic portfolio company is founded and the deal year.

### Model building

To examine the impact of VC cross-border syndication on corporate innovation, we applied negative binomial regression to test hypotheses. Equation (1) formed the econometric model to test hypothesis 1. Equation (2) and (3) are used to test hypothesis 2. Under the circumstance of β_*1*_, β_*3*_, and β_*6*_ are statistically significant, the mediation effects exist. Furthermore, perfect mediation occurs if estimated value of β_*5*_ is not statistically significant. Formally, the equations are expressed as follows:


(1) $$C\,I_{i,t}=\beta_{1}\,X_{i,t}+\beta_{2}\,C\,o\,n\,t\,r\,o\,l\,s_{i,t}+\mu_{t}+\lambda_{i}+\varepsilon_{i,t}$$

(2) $$C\,B\,Q\,R\,C_{i,t}=\beta_{3}\,X_{i,t}+\beta_{4}\,C\,o\,n\,t\,r\,o\,l\,s_{i,t}+\mu_{t}+\lambda_{i}+\varepsilon_{i,t}$$

(3) $$C\,I_{i,t}=\beta_{5}\,X_{i,t}+\beta_{6}\,C\,B\,Q\,R\,C_{i,t}+\beta_{7}\,C\,o\,n\,t\,r\,o\,l\,s_{i,t}+\mu_{t}+\lambda_{i}+\varepsilon_{i,t}$$

In both Equations (1) and (3), the explained variables are corporate innovation (*CI*_*i,t*_) measured by the number of granted invention patents, which are count variables, so this paper uses a negative binomial distribution regression model to test those two equations. In addition, since the CBQRC is a binary dummy variable, a binary discrete choice model is used when this variable is the explained variable in Equation (2). In the context of this paper, the most widely used logit and probit models of the binary discrete choice model are not applicable because the value of CBQRC is 0 in most cases and 1 in only a few cases in the entire sample of this paper. Therefore, in the binary discrete choice model, this distribution belongs to the extreme value distribution, and the model we selected to apply is the complementary log-log model.

In the above equation, *X*_*i*,*t*_refers to the explanatory variable that is the number of investment events of domestic VC cross-border syndication (*Ne*) and number of partners of domestic VC cross-border syndication (*Np*), respectively.*Controls*_*i*,*t*_ refers to a set of control variables at the VC level and firm level including the Successful exits (*Se*), IPO on foreign stock exchanges (*IPO*), VC reputation (*Rep*), State-owned background (*Sob*), VC Syndication size (*Syn*) at the VC level; and Director assignment (*Da*), CEO replacement (*CEO*), Previous corporate Innovation (*Pci*), Hi-tech dummy (*Tech*), Early dummy (*Early*), Company age (*Age*) at the firm level.

## Results

### Descriptive statistics

The descriptive statistics of each variable are shown in Table 2. According to the statistical results, the maximum and minimum values of CI are 237 and 0, respectively, indicating significant differences in the innovation capability of domestic firms. The maximum and minimum values of Ne are 163 and 0, and the maximum and minimum values of Np are 634 and 0, showing that there are significant differences in the ability of domestic VC firms to integrate into the global VC market by way of cross-border syndication. The mean value of CBQRC is 0.046, indicating that 4.6% of domestic firms will form strategic cooperation partnerships with foreign firms. The control variables are distributed in reasonable ranges.

**TABLE 2:** Summary statistics.

Variable	N	Mean value	Standard deviation	Minimum	Maximum
CI	1,311	0.775	8.244	0	237
Ne	1,311	2.479	11.240	0	163
Np	1,311	13.841	61.635	0	634
CBQRC	1,311	0.046	0.209	0	1
Se	1,311	1.107	7.039	0	91
IPO	1,311	0.295	2.432	0	41
Rep	1,311	0.074	0.262	0	1
Sob	1,311	0.291	0.455	0	1
Syn	1,311	0.223	0.416	0	1
Da	1,311	0.320	0.467	0	1
CEO	1,311	0.022	0.147	0	1
Pci	1,311	1.849	7.367	0	54
Tech	1,311	0.613	0.487	0	1
Early	1,311	0.718	0.450	0	1
Age	1,311	2.841	4.357	0	36

### Baseline results

After controlling for a series of VC level and firm level control variables, this paper empirically tests the impact of VC cross-border syndication on corporate innovation and the mediating role of CBQRC in this impact, respectively. The regression results obtained are shown in Table 3. From the regression results of Models (1) and (2), it can be found that both Ne and Np are significantly and positively associated with CI, indicating that the more cross-border syndication events of domestic VC firms, or the more cross-border syndication partners of domestic VC firms, the more beneficial to the corporate innovation. Therefore, the regression results obtained support hypothesis 1.

**TABLE 3:** Baseline regression results.

Variables	Model (1)	Model (2)	Model (3)	Model (4)	Model (5)	Model (6)
	
	CI	CI	CBQRC	CBQRC	CI	CI
Ne	0.076***		0.059***		0.034	
	(2.770)		(5.592)		(1.114)	
Np		0.013***		0.010***		0.006
		(2.951)		(6.483)		(1.213)
CBQRC					3.425***	3.374***
					(3.379)	(3.354)
Se	0.026	0.026	0.037**	0.039***	−0.018	−0.017
	(0.706)	(0.703)	(2.397)	(2.676)	(−0.402)	(−0.377)
IPO	−0.074	−0.034	0.013	0.023	−0.045	−0.027
	(−0.764)	(−0.357)	(0.175)	(0.320)	(−0.413)	(−0.257)
Rep	−1.803	−1.989*	0.935*	0.680	−1.720	−1.813*
	(−1.528)	(−1.710)	−1.914	−1.393	(−1.570)	(−1.657)
Sob	−0.206	−0.206	−0.384	−0.374	0.005	0.004
	(−0.559)	(−0.561)	(−0.895)	(−0.864)	(0.013)	(0.011)
Syn	1.969***	1.975***	0.468	0.503	2.033***	2.038***
	(4.106)	(4.126)	(1.406)	(1.505)	(4.641)	(4.651)
Da	2.073***	2.077***	0.901***	0.935***	1.342***	1.348***
	(5.887)	(5.92)	(2.886)	(2.988)	(3.708)	(3.726)
CEO	1.857	1.916	0.974	0.977	1.796	1.827
	(1.360)	(1.402)	(0.942)	(0.944)	(1.350)	(1.372)
Pci	0.049**	0.049**	0.034***	0.033***	0.055**	0.055**
	(2.176)	(2.170)	(2.805)	(2.644)	(2.388)	(2.387)
Tech	2.036***	2.043***	0.855**	0.952***	1.697***	1.703***
	(5.468)	(5.492)	(2.444)	(2.706)	(4.847)	(4.862)
Early	−1.281**	−1.285**	0.091	0.08	−1.157**	−1.159**
	(−2.371)	(−2.382)	(0.180)	(0.159)	(−2.499)	(−2.502)
Age	−0.020	−0.020	−0.012	−0.010	−0.074	−0.074
	(−0.351)	(−0.354)	(−0.252)	(−0.199)	(−1.459)	(−1.447)
Constant	−4.108***	−4.111***	−6.154***	−6.196***	−3.614***	−3.623***
	(−5.484)	(−5.492)	(−8.528)	(−8.483)	(−5.412)	(−5.420)
Year FE	Yes	Yes	Yes	Yes	Yes	Yes
Log likelihood	−596.728	−596.388	−142.53	−141.195	−588.298	−588.199
N	1,311	1,311	1,311	1,311	1,311	1,311

The values in parentheses are z-statistics. *p < 0.1, **p < 0.05, ***p < 0.01.

In addition to the main findings, we also observed some regression coefficients of the control variables in Models (1) and (2). First, in terms of control variables at the VC level: The coefficients of Se are not significant, indicating that the impact of prior successful exits of domestic VC firms on corporate innovation is not significant. The coefficients of IPO are not significant, indicating that the impact of domestic VC firms’ prior experience with IPOs on foreign exchanges on corporate innovation is not significant. The coefficients of Rep are negative, indicating that highly reputable VC firms do not better drive corporate innovation, and this result is inconsistent with the findings of Hua et al. (2016). The coefficients of Sob are not significant, indicating that there is no significant difference between state-owned background VC firms and non-state-owned background VC firms in driving corporate innovation, which is inconsistent with the findings of Bertoni and Tykvová (2015) and Pahnke et al. (2015a). The coefficients of Syn are positive and significant, indicating that VC syndication size drives corporate innovation, which is consistent with the findings of Hua et al. (2016). Second, in terms of control variables at firm level: The coefficients of Da are positive and significant, indicating that VC firms assigning directors to portfolio companies drives corporate innovation, consistent with the findings of Amornsiripanitch et al. (2019). The coefficients of CEO are insignificant, indicating that the positive effect of whether VC firms replace the CEOs of their portfolio companies with experienced external CEOs on the corporate innovation is insignificant, and this result is different from the findings of Conti and Graham (2020). The coefficients of Pci are positive and significant, indicating that the previous innovation capacity of enterprises contributes to subsequent innovation.

### The mediating effect of cross-border quadratic relationship closure

The regression results of Models (3) and (4) in Table 3 show that both Ne and Np are significantly and positively associated with CBQRC, indicating that the more cross-border syndication events of domestic VCs or the more cross-border syndication partners of domestic VCs, the greater the likelihood of establishing strategic cooperation partnership between domestic firms and foreign firms. Both Ne and Np in Models (5) and (6) are insignificantly and positively associated with CI, whereas the coefficients of CBQRC have a significant positive effect on CI at the 1% significance level, indicating that CBQRC plays a perfect mediating effect in the impact of Ne and Np on the corporate innovation, respectively. Therefore, the regression results support hypothesis 2.

To sum up, the regression results in Table 3 indicates that VC cross-border syndication can help domestic portfolio companies establish strategic cooperation partnerships with foreign portfolio companies invested by foreign VCs, thus promoting corporate innovation.

### Robustness test

To further test the robustness of the benchmark results, we adopt the instrumental variables approach and the Heckman two-step model to verify the impact of VC cross-border syndication on corporate innovation.

#### Test of endogenous problems

Due to the limited availability of some data, some important control variables may have been omitted in this paper. As previously discussed, the technical support provided by VCs to their portfolio companies (Chemmanur et al., 2014) and the incentive programs designed for innovation projects (Maas et al., 2020) are likely to influence corporate innovation, as well as the intention to collaborate between domestic firms and foreign firms (Ozmel et al., 2013b). However, since the technical support and incentive programs provided by VC firms to their portfolio companies cannot be observed, the previous benchmark regressions do not control for these two influences that may affect corporate innovation, which implies that the results of the benchmark regressions in this paper may have endogeneity problems caused by the omission of important control variables. This paper performs a two-stage regression test using the instrumental variables approach to address this issue.

The instrumental variable in this paper is VC’s prior regional investment experience (Prie), which is measured by the number of provinces (including autonomous regions and municipalities directly under the central government) in China in which VC firms have invested before investing in domestic firms. The higher the value of VC’s previous regional investment experience, the higher the degree of regional diversification of VC’s investment in China. Therefore, this instrumental variable should be positively correlated with the explanatory variables. In addition, whether VC firms can promote corporate innovation and help domestic portfolio companies establish strategic cooperation partnerships with foreign firms invested by foreign VC depends crucially on VC firms’ post-investment management strategies. Therefore, instrumental variables are not related to the explanatory or mediating variables.

Table 4 shows the regression results of the endogeneity problem test. The regression results from the first stage in Models (1) and (2), indicate that Prie are positively correlated with Ne and Np, respectively, suggesting that the instrumental variables are highly positively correlated with the explanatory variables. In addition, the values of Cragg-Donald are 128.183 and 170.410, respectively, which are much greater than the critical value of 16.38 at 10% bias, rejecting the original hypothesis of weak instrumental variables. The values of the underidentification test are 117.996 and 152.350, respectively, rejecting the original hypothesis of underidentification at the 1% level of significance. The above results suggest that VC’s prior regional investment experience (Prie) is an appropriate instrumental variable. Moreover, the regression results from the second stage in Models (3)–(8) are generally consistent with the baseline regression results, showing that the regression results continue to support hypotheses 1 and 2. Therefore, the conclusions obtained in this paper are robust after excluding possible endogenous problems.

**TABLE 4:** Robustness checks for the endogenous problems.

Variables	First stage		Second stage					
	Model (1)	Model (2)	Model (3)	Model (4)	Model (5)	Model (6)	Model (7)	Model (8)
	
	Ne	Np	CI	CI	CBQRC	CBQRC	CI	CI
Prie	0.758***	4.586***						
	(11.322)	(13.054)						
Ne			0.227**		0.059***		0.180*	
			(2.562)		(2.661)		(1.810)	
Np				0.038**		0.009**		0.030*
				(2.578)		(2.332)		(1.819)
CBQRC							5.773***	5.967***
							(2.873)	(3.114)
Controls	Yes	Yes	Yes	Yes	Yes	Yes	Yes	Yes
Year FE	Yes	Yes	Yes	Yes	Yes	Yes	Yes	Yes
Underidentification test	117.996	152.35						
Cragg-Donald	128.183	170.41						
Stock-Yogo critical value	16.38	16.38						
N	1,311	1,311	1,311	1,311	1,311	1,311	1,311	1,311

In the first-stage regression, the values in parentheses are the t-statistics of the regression coefficients, and in the second-stage regression the values in parentheses are the z-statistics. *p < 0.1, **p < 0.05, ***p < 0.01. The coefficients of the following variables are not reported due to space considerations: Se, IPO, Rep, Sob, Syn, Da, CEO, Pci, Tech, Early, Age.

#### Test of sample selection bias

Another important factor that may interfere with the reliability of the baseline regression results is the sample selection bias. Khurshed et al. (2020) found that syndication with foreign VCs will change the investment behavior of domestic VC firms, and the richer the syndication experience with foreign VC firms, the more likely domestic VC firms are to invest in high-tech or early-stage startups. Therefore, based on the findings of Khurshed et al. (2020), the baseline regression results in this paper may have a sample selection bias, that is, the experience of cross-border syndication of domestic VCs may have changed their criteria and ability to select projects that can help them choose more innovative companies as investment targets.

Potential impact of sample selection bias is controlled for by using Heckman two-stage model. First, a probit model for domestic VC firms’ choice is estimated. Second, the inverse Mills ratios (Imr) are included as an instrument in the second stage regression. The dependent variable (AI) in the probit selection model is a dummy variable that takes the value of 1 if the domestic firm in which the domestic VC firm abandoned its investment received an investment from another VC firm within the same month of the investment event, and 0 otherwise. The exogenous variable used to model domestic VC firms’ choice is the industry matching degree (Imd) between domestic VC firms and their portfolio companies. The measure of industry matching degree (Imd) is as follows: before investing in domestic firm_*A*_, the number of investment events of the industry in which VC_*B*_ invests in firm_*A*_ is divided by the total number of investment events of VC_*B*_ in China. The industry matching degree (Imd) reflects the degree of VC’s preference for the industry in which the portfolio company is located, and the larger the value of this variable, the higher the degree of VC’s preference for the industry. The results are provided in Table 5. From the first stage regression results, Imd are positive and significant in Models (1) and (2), indicating that the exogenous variable is appropriate. In second stage regression, the regression coefficients of Imr are not significant in Models (3)–(8), indicating that there is no sample selection bias, and the regression results for the other explanatory variables are generally consistent with the previous findings. Therefore, the baseline regression results in this paper are reliable.

**TABLE 5:** Robustness checks for the sample selection bias.

Variables	First stage		Second stage					
	Model (1)	Model (2)	Model (3)	Model (4)	Model (5)	Model (6)	Model (7)	Model (8)
	AI	AI	CI	CI	CBQRC	CBQRC	CI	CI
Imd	0.378***	0.378***						
	(9.727)	(9.727)						
Ne	−0.000		0.080***		0.058***		0.035	
	(−0.024)		(2.883)		(5.471)		(1.151)	
NP		−0.000		0.014***		0.010***		0.006
		(−0.028)		(3.067)		(6.413)		(1.250)
CBQRC							3.461***	3.409***
							(3.409)	(3.382)
Imr			2.348	2.357	−1.686	−1.461	2.351	2.349
			(1.368)	(1.374)	(−1.101)	(−0.952)	(1.498)	(1.496)
Controls	Yes	Yes	Yes	Yes	Yes	Yes	Yes	Yes
Year FE	Yes	Yes	Yes	Yes	Yes	Yes	Yes	Yes
Log likelihood	−6137.866	−6137.866	−595.737	−595.381	−141.707	−140.517	−587.209	−587.107
N	56,274	56,274	1,311	1,311	1,311	1,311	1,311	1,311

The values in parentheses are z-statistics. *p < 0.1, **p < 0.05, ***p < 0.01. The coefficients of the following variables are not reported due to space considerations: Se, IPO, Rep, Sob, Syn, Da, CEO, Pci, Tech, Early, Age.

## Conclusion and discussion

### Conclusion

This paper empirically investigates the impact of VC cross-border syndication on the innovation of their portfolio companies and its path of action using a sample of first-round investments in domestic firms by domestic VC firms from 2014 to 2016. The results show that (1) VC cross-border syndication has a significant positive impact on the innovation of their portfolio companies. Specifically, the more VC cross-border syndication investment events (or the more VC cross-border syndication partners), the more likely they are to promote the innovation output of their portfolio companies. (2) The CBQRC formed by four innovation agents—domestic firm—domestic VC firm—foreign VC firm—foreign firm—is the mechanism through which VC cross-border syndication affects innovation of their portfolio companies. The above findings imply that VC’s active integration into global innovation networks through cross-border syndication can help domestic firms enhance innovation capabilities in open partnerships.

### Theoretical contributions

The main contributions are as follows. First, this study proposes a new perspective to explain the impact of domestic VC cross-border syndication on domestic firm innovation. Two theories have been proposed in the previous literature to elucidate the impact of cross-border syndication by domestic VCs on themselves and their domestic portfolio companies: inter-organizational learning theory (Khurshed et al., 2020) and cross-border relationship embedding theory (Meuleman et al., 2017). Inter-organizational learning theory suggests that domestic VC can learn foreign VC’s investment skills to improve investment behavior and enhance investment performance through cross-border syndication. Cross-border relationship embedding theory suggests that if domestic VC and foreign VC have had cross-border syndication experience, they are both more likely to co-invest in domestic firms again. Compared with these two theories, we focus on the CBQRC formed by the four innovation subjects in the investment relationship, which helps to understand the impact of domestic VC cross-border syndication on themselves and domestic portfolio companies more comprehensively and deeply.

Second, this study finds a new impact mechanism for the role of domestic VCs in driving innovation in their domestic portfolio companies, which is one of the most critical issues in the field of entrepreneurship and finance. A variety of possible mechanisms have been identified in previous research, including providing technical support to portfolio companies (Chemmanur et al., 2014; Maas et al., 2020), assigning directors to portfolio companies (Amornsiripanitch et al., 2019), replacing CEOs of portfolio companies with experienced external CEOs (Conti and Graham, 2020), enhancing interaction with portfolio companies (Bernstein et al., 2016), optimizing incentive programs for innovation projects of portfolio companies (Maas et al., 2020). Unlike previous studies, this paper identifies a new impact mechanism by which domestic VC firms help domestic portfolio companies establish strategic cooperation partnerships with foreign portfolio companies invested by foreign VC firms, which are cross-border syndication partners of domestic VC firms. The discovery of this mechanism helps expand the options of strategies for VC firms to promote innovation in their portfolio companies.

### Theoretical implications

Our findings have important theoretical implications. First, VC firms can influence the innovation activities of their portfolio companies after their involvement in startups. This paper explores the relationship between VC cross-border syndication and innovation in portfolio companies, providing a new perspective for research in the context of VC internationalization. Second, this paper provides insight into the mechanisms of VC cross-border syndication that affect corporate innovation. Specifically, based on existing research, this paper finds that the CBQRC formed by domestic and foreign VCs and their respective portfolio companies affects domestic portfolio companies’ innovation. Finally, the formation of CBQRC proposed in this paper also reflects the fact that prior relationships between domestic and foreign VC firms can influence the strategic choices of portfolio companies, specifically, the linkages between VC firms of different backgrounds can serve as a bridge to guide the strategic choices of which firms to partner with.

### Managerial implications

Our findings also have significant managerial implications. First, given that the national policy-making level attaches great importance to the important role of VC in China’s innovation-driven strategy, especially in the context of gradually building a new pattern of double-cycle development, there is an urgent need to play the role of VC in supporting and catalyzing technological innovation, this paper can provide a strategic reference for domestic VC firms to enhance the innovation capability of Chinese local enterprises. Second, in the context of globalization of China’s economy and capital, domestic VC firms have also started to vigorously lay out internationalization with the intention of investing in outstanding companies globally and participating in global competition and cooperation. This paper provides an inspiration for domestic VC firms to use overseas investment to improve the innovation capability of their invested companies. Specifically, this paper focuses on the impact mechanism of VC cross-border syndication on corporate innovation, and the proposed cross-border quadratic relationship closure can also be used to explain how domestic VC firms can cultivate new advantages for China to participate in international cooperation and competition through the aggregation of capital power in the new situation.

### Limitations and future research

The CBQRC formed by the innovation subjects in the investment relationship complements the literature on the role of VC firms in their portfolio companies by showing that VC firms collect or monitor information about their portfolio companies not only for screening and monitoring purposes, but also to help companies build networks of collaborative relationships. However, limited by the data availability, this paper does not directly observe other forms of inter-firm collaboration. In the future, if we obtain other data on the exchange and interaction between domestic and foreign firms, we will further deepen and expand based on this paper to improve the completeness of our findings.

## Data availability statement

The raw data supporting the conclusions of this article will be made available by the authors, without undue reservation.

## Ethics statement

The studies involving human participants were reviewed and approved by the Northwest University Ethics Committee. Written informed consent for participation was not required for this study in accordance with the national legislation and the institutional requirements.

## Author contributions

HH created and designed the theoretical model and wrote the first manuscript. LG conceptualized the study, collected and analyzed the data, and improved the manuscript. JD contributed to the revised manuscript’s review and editing and supported scientific research funding. All authors contributed to the article and approved the submitted version.

## Conflict of interest

The authors declare that the research was conducted in the absence of any commercial or financial relationships that could be construed as a potential conflict of interest.

## Publisher’s note

All claims expressed in this article are solely those of the authors and do not necessarily represent those of their affiliated organizations, or those of the publisher, the editors and the reviewers. Any product that may be evaluated in this article, or claim that may be made by its manufacturer, is not guaranteed or endorsed by the publisher.

## Footnotes

1 PitchBook News & Analysis, 18 March 2022. Available at https://pitchbook.com/
